# Epididymal leiomyoadenomatoid tumor: A case report with literature review

**DOI:** 10.1016/j.eucr.2020.101226

**Published:** 2020-04-24

**Authors:** Bara Wazwaz, Khaled Murshed, Eiman Musa, Noheir Taha, Mohammed Akhtar

**Affiliations:** a Department of Laboratory Medicine & Pathology, Hamad Medical Corporation, Doha, Qatar; b Department of Radiology, Hamad Medical Corporation, Doha, Qatar

**Keywords:** Leiomyoadenomatoid, Epididymis, Paratesticular

## Abstract

Primary epididymal tumors are rare. Adenomatoid tumors comprise 30% of paratesticular tumors. They are benign neoplasms originating from mesothelial cells. On the other hand, leiomyomas, originating from smooth muscle cells, are less common in paratesticular region. Leiomyoadenomatoid tumor is a benign tumor which combines the morphologic features of adenomatoid tumor and leiomyoma. To the best of knowledge, only two cases of leiomyoadenomatoid tumor in the paratesticular location have been reported in the literature. We document another such case.

## Introduction

Tumors of the epididymis are rare and most are benign. Adenomatoid tumor is the most common tumor of the epididymis. It is a benign neoplasm that can also occur in other organs such as the uterus and fallopian tubes in female genital tract.^1^ Epididymal leiomyoma is the second most common tumor of the epididymis.^2^ Rarely, when a prominent smooth muscle element is found within the adenomatoid tumor, the lesion is denoted as “leiomyoadenomatoid tumor”, a term first used by Epstein.^3^ Herein, we report a leiomyoadenomatoid tumor arising in the epididymis in a 33-year old man.

## Case presentation

**Fig. 1:**
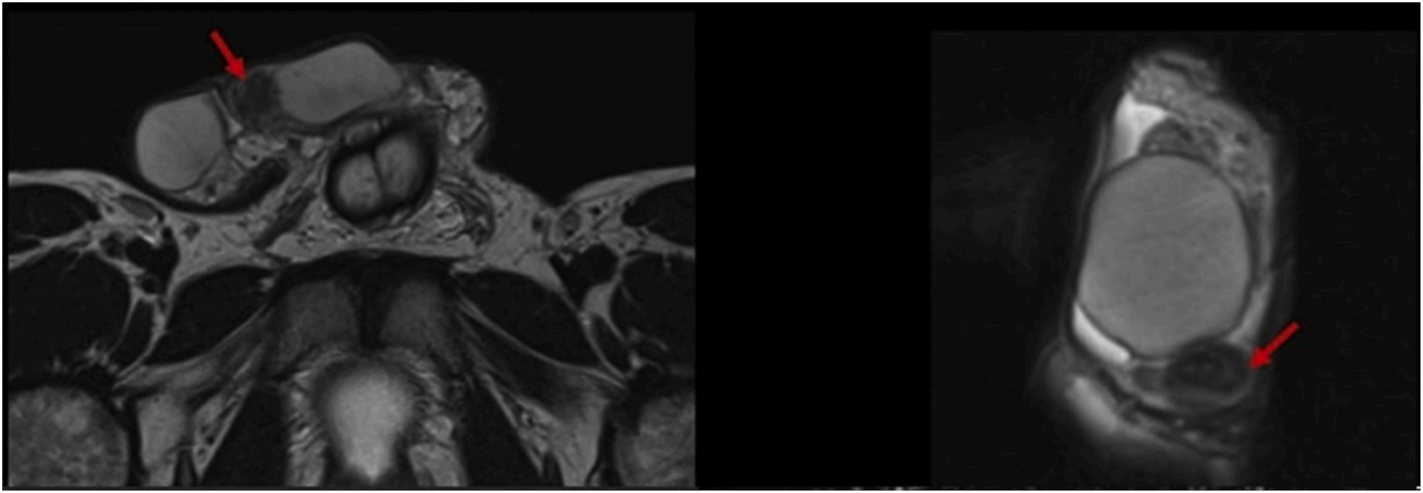
Scrotal MRI: the extra testicular mass appears in relation to the left epididymis tail and display very low T2 signal relative to testicular parenchyma.

A previously healthy 33-year-old male patient presented with left painless scrotal swelling for the past 2 years, which increased in size slightly in the past year. He had no other symptoms. Physical examination showed scrotal hard round nodule measuring around 15 mm. Routine laboratory tests including complete blood count, blood biochemistry, and urinalysis were within normal limits. Scrotal ultrasonography revealed a well-defined heterogeneous predominantly hypoechoic lesion in the left epididymis measuring 14 × 12 mm with internal vascularity. Scrotal MRI showed left extra-testicular solid mass in relation to the epididymis tail measuring 13 × 13 mm with very low T2 signal intensity (Fig. 1).

Subsequently, left scrotal exploration revealed a mass noticed related to the lower pole of the testis, likely from epididymis tail. Tunica eversion was done, and the mass was dissected from surroundings. The patient's postoperative course was uneventful. On gross examination, the specimen consisted of 13 × 13 mm tan brown firm encapsulated mass. Cut surfaces were firm, white and homogenous.

**Fig. 2:**
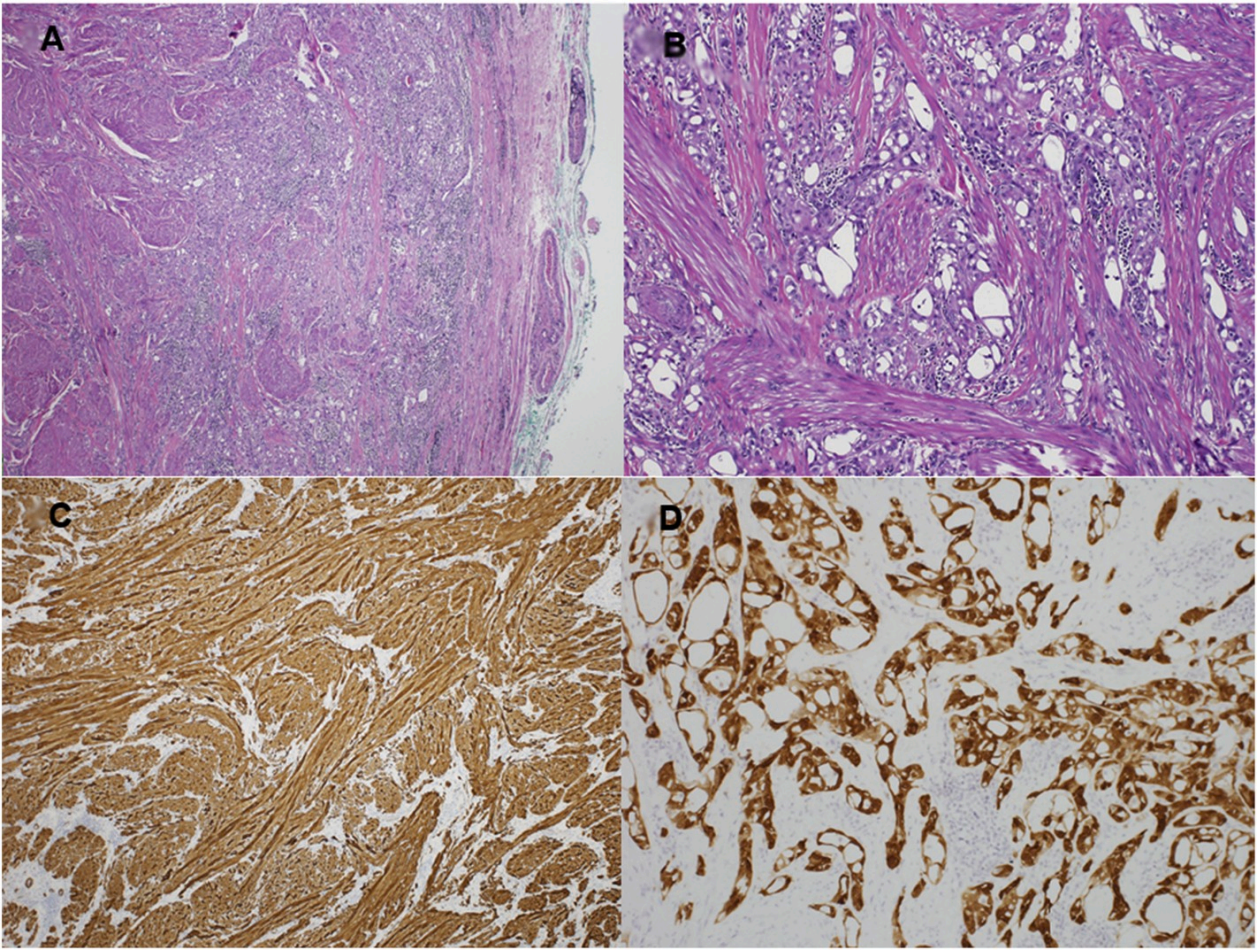
Microscopic and immunohistochemical features of the tumor: **A**, photomicrograph depicting a well-defined biphasic neoplasm (Haematoxylin & Eosin stain, x40). **B**, high power view shows that the adenomatoid gland-like component intermingles with spindle smooth muscle component (H&E stain, x100). C, fascicles of spindle smooth muscle cells are reactive for Caldesmon antibody (IHC, x40). **D**, the gland-like adenomatoid component is positive for Calretinin antibody (IHC, x100).

Microscopically, the tumor was well-defined, composed of two components; the first predominant component comprised fascicles of spindle smooth muscle proliferation, intermingled with a second component composed of gland-like spaces lined by flat to cuboidal cells having round nuclei and conspicuous nucleoli (Fig. 2). No nuclear atypia, mitotic activity or necrosis were identified.

Immunohistochemical studies showed fascicles of spindle smooth muscle cells stained by Caldesmon and Smooth Muscle Actin (SMA). The gland-like component showed reactivity for Calretinin, Cytokeratin 7, Cytokeratin AE1/AE3 and Podoplanin, but stained negative for Cytokeratin 5/6. The two components observed; mesothelial and muscular, lead to the diagnosis of a leiomyoadenomatoid tumor.

## Discussion

Leiomyoadenomatoid tumor is an extremely rare benign neoplasm of the epididymis that have biphasic pattern: an adenomatoid component intermixed with fascicles of smooth muscle cells. There is no agreement on the pathogenesis of this exceedingly rare entity.

It is essential to mention that an exuberant growth of smooth muscle component can sometimes obscure the adenomatoid epithelial-like component, which may result in misdiagnosis of malignant tumor infiltrating a smooth muscle bundles.

This entity has also been described to occur in the female genital tract, most commonly arising from the uterus. To our knowledge, our case is the third documented epidydimal leiomyoadenomatoid tumor (Table 1).^4^^,^^5^ The previous cases reported the tumor in the right epididymis, whereas this case was in the left side. Furthermore, the size of tumor in our case was smaller, and the patient was younger. The case seems to be treated safely with local excision and no recurrence was reported. Our patient will be followed for any unexpected development.

**Table 1:** Cases of epididymal leiomyoadenomatoid tumor.

Author	Year	Patient's age	Tumor size	Tumor laterality	Radiological features
**Kausch et al**^**4**^	2002	44	30 x 20 mm	Right	Ultrasonography: Hypodense and hyperdense mass in the right epididymal tail
**Cazorla et al**^**5**^	2014	57	20 x 15 mm	Right	Ultrasonography: Extra-testicular well-limited, heterogeneous, and mainly hypoechogenic mass
**Current case**	2020	33	13 x 13 mm	Left	MRI: Extra- testicular mass appears in relation to the left epididymis tail and display very low T2 signal relative to testicular parenchyma

## Conclusion

Leiomyoadenomatoid tumor is a very rare benign neoplasm of the epididymis that is characterized by having two components; adenomatoid and smooth muscle. We are presenting the third case of epididymal leiomyoadenomatoid tumor to familiarize clinicians and pathologists about this exceedingly rare entity.

## Declaration of competing interest

The authors declared no potential conflicts of interest.
